# Acidocalcisome-Mitochondrion Membrane Contact Sites in *Trypanosoma brucei*

**DOI:** 10.3390/pathogens7020033

**Published:** 2018-03-22

**Authors:** Srinivasan Ramakrishnan, Beejan Asady, Roberto Docampo

**Affiliations:** 1 Center for Tropical and Emerging Global Diseases, University of Georgia, Athens, GA 30602, USA; sri@uga.edu (S.R.); basady@uga.edu (B.A.); 2 Department of Cellular Biology, University of Georgia, Athens, GA 30602, USA

**Keywords:** membrane contact sites, calcium signaling, trypanosomes

## Abstract

Membrane contact sites are regions of close apposition between two organelles, typically less than 30 nanometers apart, that facilitate transfer of biomolecules. The presence of contact sites has been demonstrated in yeast, plants, and mammalian cells. Here, we investigated the presence of such contact sites in *Trypanosoma brucei*. In mammalian cells, endoplasmic reticulum-mitochondria contact sites facilitate mitochondrial uptake of Ca^2+^ released by the ER-located inositol 1,4,5-trisphosphate receptor (InsP_3_R). However, the InsP_3_R in trypanosomes localizes to acidocalcisomes, which serve as major Ca^2+^ stores in these parasites. In this work, we have used super-resolution structured illumination microscopy and electron microscopy to identify membrane contact sites that exist between acidocalcisomes and mitochondria. Furthermore, we have confirmed the close association of these organelles using proximity ligation assays. Characterization of these contact sites may be a necessary starting point towards unraveling the role of Ca^2+^ in regulating trypanosome bioenergetics.

## 1. Introduction

*Trypanosoma brucei* has two life cycle stages that are easily grown in the laboratory, the procyclic form (PCF), which is one of the stages present in the insect vector, and the bloodstream form (BSF), which is similar to the form present in the blood of the mammalian host. The PCF’s mitochondria are well developed and possess a respiratory chain while the BSF’s mitochondria are more rudimentary and do not have a functional respiratory chain nor can they perform oxidative phosphorylation; however, the mitochondria of both stages maintain a membrane potential [1,2,3,4] and are able to transport Ca^2+^ [2].

In vertebrate cells, the constitutive inositol 1,4,5-trisphosphate receptor (InsP_3_R)-dependent Ca^2+^ transfer from the endoplasmic reticulum (ER) to the mitochondria has been found to be an essential cellular process required for efficient mitochondrial respiration and maintenance of normal cell bioenergetics [5]. This role is facilitated by the close apposition of the ER and mitochondria supported by physical linkages, or membrane contact sites (MCS). This apposition creates microdomains of high Ca^2+^ concentration that facilitate rapid Ca^2+^ transport into the mitochondria [6,7,8,9] through the mitochondrial Ca^2+^ uniporter (MCU) [10,11].

In trypanosomatids the InsP_3_R is located in acidocalcisomes instead of the ER [12,13]. Acidocalcisomes are acidic calcium stores that appear to play a fundamental role in Ca^2+^ signaling in these eukaryotes [14]. Previous research has demonstrated that release of Ca^2+^ from acidocalcisomes causes an eight-fold increase in mitochondrial Ca^2+^ concentration but only a two-fold increase in the cytosolic Ca^2+^ concentration [15], suggesting a role of the MCU complex (MCUC) of trypanosomes in shaping the amplitude and spatiotemporal patterns of cytosolic Ca^2+^ increases [16]. These results also suggest a very close proximity between acidocalcisomes and the sole mitochondrion of *T. brucei* and that a similar constitutive InsP_3_R-dependent Ca^2+^ release from acidocalcisomes [12] could be involved in the regulation of cell bioenergetics and cell death, as occurs between the ER and mitochondria of vertebrate cells [5].

In this work, we explored the presence of MCSs between these organelles using high-resolution microscopy, electron microscopy and the proximity ligation assay, a technique that is specialized for visualizing contact sites [17,18,19], and demonstrate that the membrane of acidocalcisomes indeed contact the mitochondrial outer membrane. We show that several of these described contacts occur within each cell in both PCF and BSF of *T. brucei*. Our findings lay the foundation for investigating acidocalcisome-mitochondrion Ca^2+^ transfer and will also stimulate further research in the search for contact sites between other organelles in trypanosomatid parasites.

## 2. Results 

**Figure 1:**
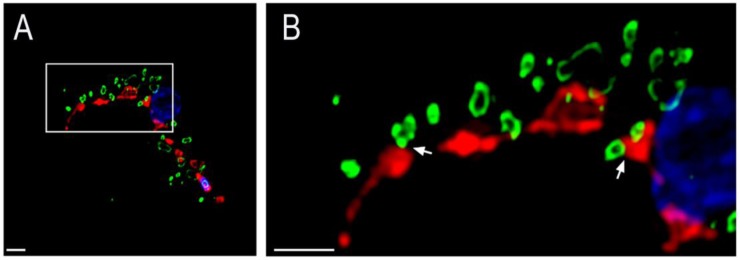

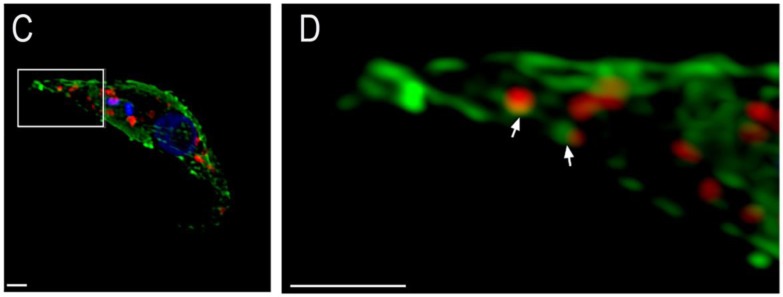
Representative super resolution images of *T. brucei* acidocalcisomes and mitochondria in BSF (**A**,**B**) and procyclic form (PCF) (**C**,**D**) trypanosomes. Close proximity between the acidocalcisomes and mitochondria could be observed in both life cycle stages and are indicated by white arrows in the magnified images (**B**,**D**). Mitochondria are in red in A/B and in green in C/D. Acidocalcisomes are in green in A/B and in red in B/C. DAPI staining of nuclei and kinetoplasts is in blue. Scale bar = 1 µm.

**Figure 2:**
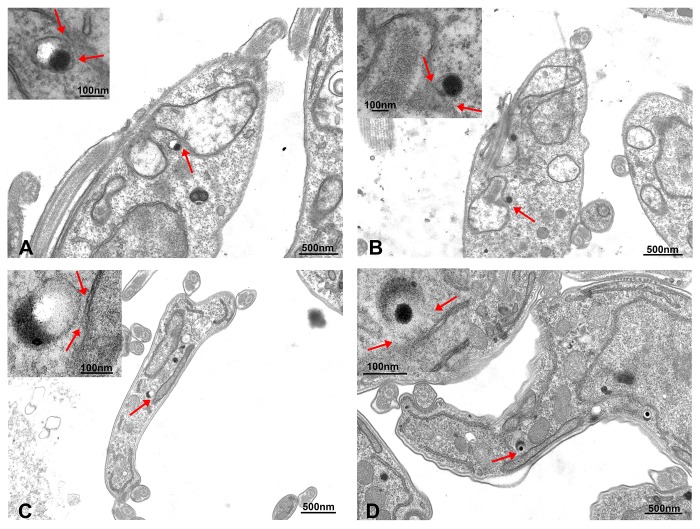
Representative transmission electron microscopy images of PCF (**A**,**B**) and bloodstream form (BSF) (**C**,**D**) *T. brucei* showing contacts between acidocalcisomes and the mitochondrion of the parasites. Acidocalcisomes appear as rounded organelles containing electron-dense material that adheres to one side of the membrane, and are seen adjacent to the mitochondrion double membrane. The contact sites could be observed in both life cycle forms and are indicated by red arrows in the insets at higher magnification.

Since acidocalcisomes are the main Ca^2+^ storage site in trypanosomes [14] and possess an InsP_3_R [12], we hypothesized that they could be in close proximity to *T. brucei* mitochondrion, allowing rapid Ca^2+^ transfer across the organellar membranes. To test this hypothesis, we expressed a *T. brucei* mitochondrial outer membrane protein, the voltage-dependent anion channel (TbVDAC) [20,21], tagged with 3xHA, to label this membrane using anti-HA antibodies. Next, we used the *T. brucei* vacuolar H^+^-pyrophosphatase 1 (TbVP1) antibody to label the *T. brucei* acidocalcisome membrane [22]. Using super-resolution structured illumination microscopy we noticed that acidocalcisomes are often located in close vicinity to the mitochondrion. We performed such analyses in both PCF and BSF trypanosomes. In both life cycle stages, we could observe a close association between acidocalcisomes and the mitochondrion of the parasite (Figure 1). Furthermore, such contact sites could be observed in both life cycle stages wild type forms using electron microscopy as well (Figure 2). In these electron micrographs, it can be observed that in all such cases of contact, the membranes of the two organelles appear to be less than 30 nm apart in both PCF (19.4 ± 6.2 nm in *n* = 3) and BSF (23.2 ± 4.3 nm in *n* = 3).

**Figure 3:**
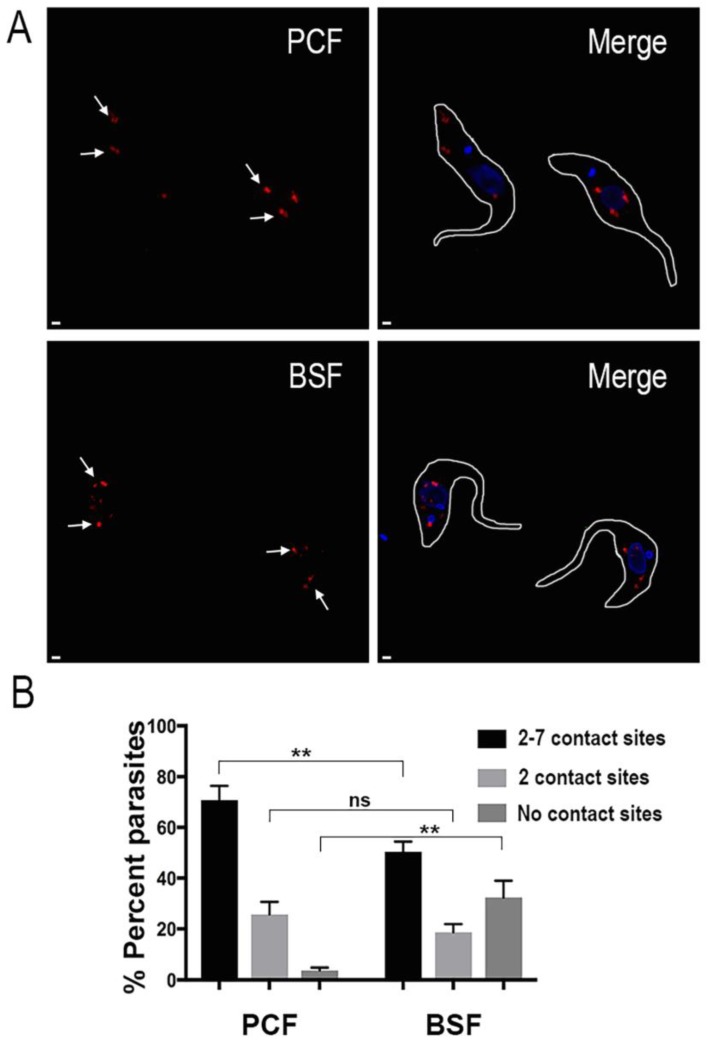
(**A**) Representative super resolution structured illumination images of *T. brucei* PCF and BSF trypanosomes subjected to proximity ligation assay. The red fluorescent signals indicated by the white arrows confirm the existence of membrane contact sites between acidocalcisomes and the mitochondrion in these parasites. Only some of them are labeled with arrows. DAPI staining is in blue. Merge images are at right. Scale bars = 1 µm; (**B**) Quantification of the number of contact sites observed using the proximity ligation assay. A total of one hundred PCF or one hundred BSF parasites were counted in each of three independent biological experiments (*n* = 3) and classified according to the number of contact sites observed in them. Values shown are means ± s.d. of *n* = 3. ** *p* < 0.05, Student’s *t* test; ns is no significant difference.

To further characterize such points of contact we performed a proximity ligation assay (PLA). Fixed and permeabilized parasites were treated with primary antibodies to label the mitochondrial outer membrane and the acidocalcisomes. Then, the cells were subjected to PLA analysis. Following this method, the points of close contact between the two organelles can be observed as red fluorescent spots (Figure 3A, marked by white arrows). Acidocalcisome-mitochondrion membrane contact sites could be clearly observed in both PCF and BSF parasites. Interestingly, we found that acidocalcisome-mitochondrion membrane contact sites are more prevalent in PCF trypanosomes. Nevertheless, a substantial number of contact sites were observed in both life cycle stages (Figure 3B). A negative control using a mitochondrial inner membrane protein (mitochondrial calcium uniporter or MCU) tagged with 3xHA [23] instead of TbVDAC-3xHA, did not result in any labeling (data not shown).

## 3. Discussion 

Ca^2+^ signaling regulates important processes in all organisms [24,25,26]. As a result, tight regulation of Ca^2+^ signaling is necessary in all cell types. This tight regulation is facilitated by the action of several Ca^2+^ binding proteins and transporters, which have been identified and characterized previously [27]. However, a role for MCSs in Ca^2+^ signaling and regulation is now emerging. In mammalian cells such MCSs are the main facilitators of Ca^2+^ entry into the cell [28]. Furthermore, MCSs also participate in mitochondrial Ca^2+^ import and thereby regulate the energy production and cell death processes within cells.

Our work is the first reporting MCSs in trypanosomes. Using immunofluorescence and super resolution structured illumination microscopy, as well as electron microscopy, we show that acidocalcisomes, the acidic Ca^2+^ stores of *T. brucei*, reside in close vicinity to the mitochondrion. To validate this observation, we used the proximity ligation assay, a tool specially designed to detect MCSs [17,18,19]. Using this tool we demonstrated that such MCSs are found in both PCF and BSF of *T. brucei*.

Overexpression of TbVDAC could have affected the extent of the contact sites. However, contact sites were also detected by electron microscopy of wild type cells.

**Figure 4:**
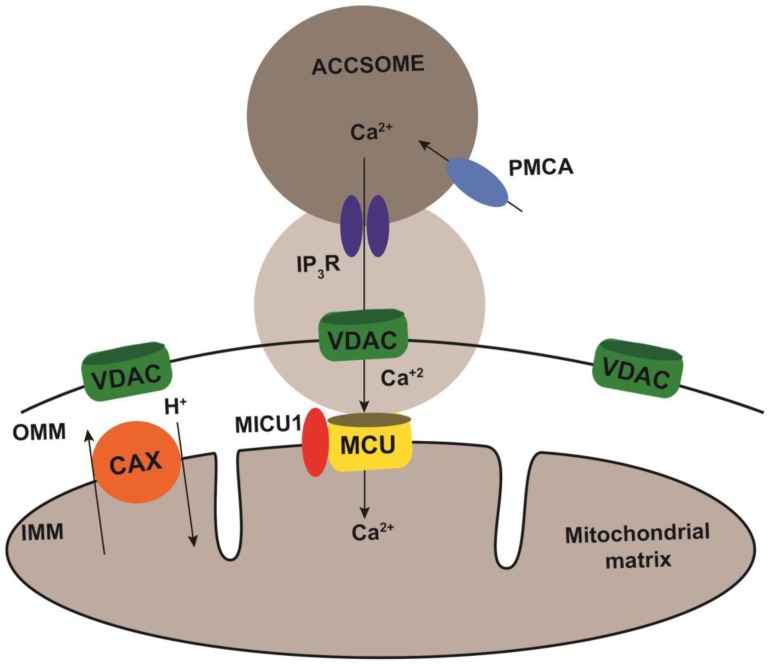
Model showing acidocalcisome-mitochondrion contact sites and their putative role in Ca^2+^ transfer in the kinetoplastid parasite *T. brucei*. The scheme shows the mitochondrial Ca^2+^ uniporter (MCU), and its gatekeeper, mitochondrial calcium uptake 1 (MICU1), and the Ca^2+^/H^+^ exchanger mediating Ca^2+^ efflux across the inner mitochondrial membrane (IMM). The voltage-dependent anion channel (VDAC) is in the outer mitochondrial membrane (OMM). The acidocalcisome (Accsome) shows the inositol 1,4,5-trisphosphate receptor channel (IP_3_R) for Ca^2+^ release and plasma membrane-type Ca^2+^-ATPase (PMCA) for Ca^2+^ uptake. The grey area between the acidocalcisome and mitochondrion represents a Ca^2+^ microdomain.

Based on our observations we propose the model illustrated in Figure 4. The close apposition of acidocalcisomes to the mitochondrial outer membrane would create microdomains of high Ca^2+^ concentration generated by InsP_3_R-driven Ca^2+^ release, which could be rapidly transferred to the mitochondrion through the VDAC and the MCUC. It is interesting to note that the phosphoinositide-phospholipase C (PI-PLC) of *T. brucei*, the enzyme responsible for InsP_3_ generation, is active at physiological Ca^2+^ concentrations, suggesting its constitutive activation [29]. Constitutive release of InsP_3_ would lead to acidocalcisome Ca^2+^ release and transfer to the mitochondrion regulating the bioenergetics of the cells, as it has been described in mammalian cells for the role of the ER-mitochondria contact sites [5].

MCSs are often held together by tethering proteins [30]. Close contacts between ER and mitochondria in yeast are maintained by the ER-mitochondria encounter structure (ERMES), which is absent in mammalian cells [31], except for the SMP domain of PDZD8 that is a functional ortholog to the SMP domain of yeast Mmm1 [32]. Orthologs to Mmm2 and Mdm12 have also been proposed in *T. brucei* [33] although they have not beed studied in detail.

In mammalian cells, ER-mitochondria MCSs are tethered by the protein GRP75 [34] and other proteins [35]. However, even if orthologs to these proteins were present in trypanosomes there is no reason to believe that they would be involved in maintaining acidocalcisome-mitochondrion MCSs. Further work is needed to investigate the specific tethers involved in this process. In mammalian cells, MCSs have been reported between multiple organelles. The ER, being the largest membrane-bound organelle, plays a main role in forming MCSs. ER-plasma membrane, ER-Golgi, ER-endosome, and ER-lipid droplet MCSs with specific functions have been described in mammalian cells [36]. Other inter-organellar MCSs such as mitochondria-vacuole [37,38], nucleus-vacuole [39], and mitochondria–lipid droplet [40] also seem to have specific intracellular roles. Indeed, it is possible that such MCSs between other organelles also exist in *T. brucei*. Using techniques such as proximity ligation assay and super-resolution microscopy or electron microscopy analysis, the existence of such organellar MCSs can be investigated further in *T. brucei*. In this regard, previous work in *T. cruzi* has shown that acidocalcisomes are frequently seen in close contact with other organelles and intracellular structures (mitochondria, nucleus, lipid inclusions and subpellicular microtubules) [41]. Such studies would help identify new proteins and mechanisms underlying the biology of *T. brucei*.

## 4. Materials and Methods 

### 4.1. Culture Methods 

*T. brucei* PCF 29-13 and BSF single marker strains (gifts from Dr. George A.M. Cross, Rockefeller University, New York) were used for all our experiments. PCF trypanosomes were grown at 27 °C in SDM-79 medium [42], and supplemented with hemin (7.5 µg/mL) and 10% heat-inactivated tetracycline-free fetal bovine serum. BSF trypanosomes were grown at 37 °C in HMI-9 medium [43], supplemented with 10% tetracycline-free fetal bovine serum (FBS).

### 4.2. Plasmids, Chemicals and Reagents

The pLEW100v5-BSD vector was a gift from Dr. George A.M. Cross (Rockefeller University). TbVDAC was amplified from *T. brucei* genomic DNA using the forward primer–GTACGGATCCTTAAGCGTAATCTGGAACATCGTATGGGTAAGCGTAATCTGGAACATCGTATGGGTAAGCGTAATCTGGAACATCGTATGGGTACGAATGGGTAATAAGGAC and reverse primer-GTACAAGCTTATGGGTCCCAGTGAGTAC and Q5 2X mastermix (Cat# M0492S) from New England Biolabs (Ipswich, MA, USA). The amplified fragment was cloned using BamHI and HindIII restriction sites into the pLEW100v5-BSD plasmid and sequenced before transfection into the parasites. Rabbit polyclonal antibody against HA (ab9110) was purchased from Abcam (Cambridge, UK). Mouse antibodies against the vacuolar proton pyrophosphatase (TbVP1) were reported previously [22] and were provided by Dr. Norbert Bakalara (University of Montpellier, Montpellier, France). Alexa-conjugated secondary antibodies were purchased from Invitrogen (Thermo Fisher Scientific, Carlsbad, CA, USA). AMAXA human T-cell Nucleofector kit was purchased from Lonza (Basel, Switzerland). The primers were purchased from Integrated DNA Technologies (Coralville, IA, USA). Duolink Proximity ligation assay kit (DUO92101), and all other reagents of analytical grade were purchased from Sigma-Aldrich (St. Louis, MO, USA).

### 4.3. Preparation of Guinea Pig Antibody against T. brucei Vacuolar Pyrophosphatase (TbVP1)

A 357-bp fragment comprising the loop III and adjacent regions of *T. bruce*i VP1 gene was amplified and cloned into pET23a, as previously described [22]. This construct was transformed into *E. coli* BL21-CodonPlus (Agilent Technologies, Santa Clara, CA, USA) for expression. Cells were lysed, and the recombinant protein was purified using HisPurNi-NTA columns under denaturing conditions, as described by the manufacturer (Thermo Fisher Scientific, Walthman, MA, USA). Antibodies were produced in Hartley strain Guinea pigs (Charles River Laboratories), weighing about 600 g each. Antigen was delivered by subdermal injections while the animal was under anesthesia with isofluorane. The primary inoculation contained 200 µg purified protein mixed in equal parts with Freund’s complete adjuvant. Two subsequent boosts, spaced in 3 week intervals, contained 100 µg purified protein mixed in equal parts with Freund’s incomplete adjuvant. Final bleeds were collected via cardiac puncture, while the animal was under deep anesthesia with isofluorane. This antibody was previously shown to localize to acidocalcisomes [44]. The study was carried out in strict accordance with the recommendations in the Guide for the Care and Use of Laboratory Animals of the United States National Institutes of Health. The animal protocol was approved by the Institutional Animal Care and Use Committee of the University of Georgia.

### 4.4. Cell Transfection

Cell transfections were done as reported previously [45]. In brief, mid-log phase PCF trypanosomes (5 × 10^6^ cells/mL) were harvested by centrifugation at 1000× *g* for 10 min, washed with a Cytomix buffer (2 mM EGTA, 3 mM MgCl_2_, 120 mM KCl, 0.5% glucose, 0.15 mM CaCl_2_, 0.1 mg/mL BSA, 10 mM K_2_HPO_4_/KH_2_PO_4_, 1 mM hypoxanthine, 25 mM Hepes, pH 7.6) and resuspended in 0.4 mL of the same buffer at a cell density of 2.5 × 10^7^ cells/mL. The washed cells were mixed with 50 µg of plasmid DNA in a 0.4-cm electroporation cuvette and subjected to two pulses from a Bio-Rad Gene Pulser Xcell (Bio-Rad, Hercules, CA, USA) electroporator set at 1.5 kV and 25 µF. The stable transformants were obtained in SDM-79 medium supplemented with 15% FBS and 10 µg/mL blasticidin. For BSF trypanosomes, 10 µg of NotI-linearized plasmid DNA was used per 4 × 10^7^ mid-log phase cells in 100 µL AMAXA Human T-cell Nucleofector solution. Electroporation was performed using 2 mm gap cuvettes with program X-001 of the AMAXA Nucleofector. Following each transfection, stable transformants were selected and cloned by limiting dilution in HMI-9 medium containing 15% FBS with 5 µg/mL blasticidin in 24-well plates. The correct expression and regulation of epitope-tagged VDAC was confirmed by immunofluorescence and western blot analyses in absence or presence of 1 µg/mL tetracycline in the culture medium.

### 4.5. Immunofluorescence Analyses

PCF or BSF trypanosomes were washed with Buffer A with glucose (BAG, 116 mM NaCl, 5.4 mM KCl, 0.8 mM MgSO_4_, 50 mM Hepes, pH 7.2, and 5.5 mM glucose) and then fixed with 4% paraformaldehyde at room temperature (RT) for 1 h. The fixed parasites were washed twice with PBS for 10 min and then were allowed to adhere to poly-l-lysine-coated coverslips and permeabilized with 0.5% Triton X-100/PBS for 3 min for PCF and 0.1% Triton X-100/PBS for 5 min for BSF. After blocking with PBS containing 3% BSA, 1% fish gelatin, 50 mM NH_4_Cl and 5% goat serum for 1 h, trypanosomes were stained in 3% BSA/PBS with the guinea pig antibody against TbVP1 (1:500) and anti-HA rabbit polyclonal antibody against HA (1:3500). After thoroughly washing with PBS, cells were incubated with Alexa 488 or 546-conjugated goat anti-rabbit antibody or goat anti-guinea pig at 1:1000 for 1 h. The cells were counterstained with DAPI before mounting with Gold ProLong Gold antifade reagent (Molecular Probes) (Thermo-Scientific, Waltham, MA, USA). Fluorescent optical images were taken with a 100× oil immersion objective, a high-power solid-state 405 nm laser and EM-CCD camera (Andor iXon) (Andor Technology Ltd., Belfast, UK) under nonsaturating conditions in a Zeiss ELYRA S1 (SR-SIM) super resolution microscope. Images were acquired and processed with ZEN 2011 software with SIM analysis module. The ELYRA S1 system achieves a lateral resolution of ~100 nm and an axial resolution of ~200 nm through the use of SR-SIM.

### 4.6. Transmission Electron Microscopy

Mid-log phase PCF and BSF *T. brucei* were fixed overnight at RT in 2.5% glutaraldehyde in 0.1 M cacodylate-HCl buffer, pH 7.2. The fixed parasites were rinsed in buffer and enrobed in 4% noble agar. Parasites were post-fixed for 30 min with 1% OsO_4_, 1.25% potassium ferrocyanide, and 5 mM CaCl_2_ in 0.1 M cacodylate buffer, pH 7.2, dehydrated in an ascending ethanol series, and embedded in Epon resin. Ultrathin sections were stained with uranyl acetate and lead citrate. Images were acquired at 80 kV using a JEOL JEM 1011 transmission electron microscope equipped with an XR80M Wide-Angle Multi-Discipline Mid-Mount CCD Camera from AMT (Advanced Microscopy Techniques) (AMT corp., Woburn, MA, USA).

### 4.7. Proximity Ligation Assay

Proximity ligation assay was performed according to the manufacturer’s instructions. Briefly, PCF or BSF trypanosomes were washed with BAG and then fixed with 4% paraformaldehyde at RT for 1 h. The fixed parasites were washed twice with PBS for 10 min and then were allowed to adhere to poly-l-lysine-coated coverslips and permeabilized with 0.5% Triton X-100/PBS for 3 min for PCF and 0.1% Triton X-100/PBS for 5 min for BSF. After blocking with PBS containing 3% BSA, 1% fish gelatin, 50 mM NH_4_Cl and 5% goat serum for 1 h, trypanosomes were stained in 3% BSA/PBS with the mouse antibody against TbVP1 (1:500) and rabbit polyclonal antibody against HA (1:3500). A 1% bovine serum albumin solution containing the mouse and rabbit probes from the duolink kit was prepared and incubated at RT for 20 min. After thoroughly washing the coverslips with PBS, they were incubated in the solution containing the rabbit and mouse probes for 1 h at 37 °C. Next, the coverslips were washed twice with buffer A provided with the kit and incubated at 37 °C for 30 min with the Duolink Ligase in the Duolink ligation buffer. Next, the coverslips were washed twice with buffer A provided with the kit and then incubated at 37 °C for 100 min with the Duolink polymerase in the Duolink amplification buffer. Lastly, the coverslips were washed with buffer B and mounted on Duolink in situ mounting media with DAPI. Fluorescent optical images were taken with a 100× oil immersion objective, a high-power solid-state 405 nm laser and EM-CCD camera (Andor iXon) under nonsaturating conditions in a Zeiss ELYRA S1 (SR-SIM) super resolution microscope. Images were acquired and processed with ZEN 2011 software with SIM analysis module.

### 4.8. Statistical Analysis 

All values are expressed as means ± s.d. and *n* refers to the number of independent biological experiments performed. Statistical analyses were conducted using GraphPad Prism 5 (GraphPad Software, San Diego, CA, USA).
